# Dual RNA sequencing of *Helicobacter pylori* and host cell transcriptomes reveals ontologically distinct host-pathogen interaction

**DOI:** 10.1128/msystems.00206-24

**Published:** 2024-03-22

**Authors:** Wei Hu, Zhi Yong Zhai, Zhao Yu Huang, Ze Min Chen, Ping Zhou, Xia Xi Li, Gen Hua Yang, Chong Ju Bao, Li Juan You, Xiao Bing Cui, Gui Li Xia, Mei Ping Ou Yang, Lin Zhang, William Ka Kei Wu, Long Fei Li, Yu Xuan Zhang, Zhan Gang Xiao, Wei Gong

**Affiliations:** 1 Department of Gastroenterology, Shenzhen Hospital, Southern Medical University, Shenzhen, Guangdong, China; 2 The Third School of Clinical Medicine, Southern Medical University, Shenzhen, Guangdong, China; 3 Department of Anaesthesia and Intensive Care and Peter Hung Pain Research Institute, The Chinese University of Hong Kong, Hong Kong, China; 4 Department of Medicine and Therapeutics, The Chinese University of Hong Kong, Hong Kong, China; 5 Guangdong Engineering Technology Research Center of Reproductive Immunology for Peri-implantation, Shenzhen Key Laboratory of Reproductive Immunology for Peri-implantation, Shenzhen Zhongshan Institute for Reproduction and Genetics, Shenzhen Zhongshan Urology Hospital, Shenzhen, Guangdong, China; 6 Department of Pharmacology and Therapeutics, King’s College London, London, United Kingdom; 7 Laboratory of Molecular Pharmacology, Department of Pharmacology, School of Pharmacy, Southwest Medical University, Luzhou, China; 8 South Sichuan Institute of Translational Medicine, Luzhou, China; 9 Laboratory of Personalized Cell Therapy & Cell Medicines, School of Pharmacy, Southwest Medical University, Luzhou, China; Northern Arizona University, Flagstaff, Arizona, USA

**Keywords:** *Helicobacter pylori*, cytotoxin-associated genes A, dual RNA sequencing, ATP-binding cassette transporter, oxidative phosphorylation, alternative splicing, inflammatory bowel disease

## Abstract

*Helicobacter pylori* is a highly successful pathogen that poses a substantial threat to human health. However, the dynamic interaction between *H. pylori* and the human gastric epithelium has not been fully investigated. In this study, using dual RNA sequencing technology, we characterized a cytotoxin-associated gene A (*cagA*)-modulated bacterial adaption strategy by enhancing the expression of ATP-binding cassette transporter-related genes, *metQ* and *HP_0888*, upon coculturing with human gastric epithelial cells. We observed a general repression of electron transport-associated genes by *cagA*, leading to the activation of oxidative phosphorylation. Temporal profiling of host mRNA signatures revealed the downregulation of multiple splicing regulators due to bacterial infection, resulting in aberrant pre-mRNA splicing of functional genes involved in the cell cycle process in response to *H. pylori* infection. Moreover, we demonstrated a protective effect of gastric *H. pylori* colonization against chronic dextran sulfate sodium (DSS)-induced colitis. Mechanistically, we identified a cluster of propionic and butyric acid-producing bacteria, *Muribaculaceae*, selectively enriched in the colons of *H. pylori*-pre-colonized mice, which may contribute to the restoration of intestinal barrier function damaged by DSS treatment. Collectively, this study presents the first dual-transcriptome analysis of *H. pylori* during its dynamic interaction with gastric epithelial cells and provides new insights into strategies through which *H. pylori* promotes infection and pathogenesis in the human gastric epithelium.

**IMPORTANCE:**

Simultaneous profiling of the dynamic interaction between *Helicobacter pylori* and the human gastric epithelium represents a novel strategy for identifying regulatory responses that drive pathogenesis. This study presents the first dual-transcriptome analysis of *H. pylori* when cocultured with gastric epithelial cells, revealing a bacterial adaptation strategy and a general repression of electron transportation-associated genes, both of which were modulated by cytotoxin-associated gene A (*cagA*). Temporal profiling of host mRNA signatures dissected the aberrant pre-mRNA splicing of functional genes involved in the cell cycle process in response to *H. pylori* infection. We demonstrated a protective effect of gastric *H. pylori* colonization against chronic DSS-induced colitis through both *in vitro* and *in vivo* experiments. These findings significantly enhance our understanding of how *H. pylori* promotes infection and pathogenesis in the human gastric epithelium and provide evidence to identify targets for antimicrobial therapies.

## INTRODUCTION

*Helicobacter pylori*, a Gram-negative and transmissible pathogen that colonizes the stomachs of almost half of the world’s population, poses a substantial threat to human health (1). The World Health Organization has classified *H. pylori* as a “high-priority” pathogen (2). According to the consensus of the 2015 Kyoto conference, all *H. pylori*-positive individuals should receive eradication therapy unless there are compelling contraindications (3). Patients infected with this pathogen typically exhibit a sustained immune response (chronic gastritis) that can progress to atrophic gastritis, intestinal metaplasia (IM), and, ultimately, gastric adenocarcinoma, according to Correa’s cascade (4). Studies have determined that several virulence factors contribute to the pathogenicity of *H. pylori*, especially the cytotoxin-associated gene A (*cagA*), which is delivered through bacterial type IV secretion and causes chronic inflammation and oncogenesis in gastric epithelial cells (5).

*H. pylori* was initially identified as a noninvasive microorganism adhering to the gastric mucosa and surviving in the gastric lumen. However, its facultative intracellular nature has been increasingly recognized by researchers in recent decades, with evidence revealing that the bacterium can invade, survive, and replicate in both epithelial cells and professional phagocytes *in vitro* and *in vivo* (6–8). A small proportion of *H. pylori* was found to colonize within parietal cells in the murine gastric epithelium (9). This ability to reside within gastric epithelial cells endows *H. pylori* with a selective survival advantage, allowing it to evade eradication by immunocytes and antibiotics that act extracellularly. Capurro et al. reported that *H. pylori* secretes vacuolating cytotoxin A (*vacA*), a virulence factor, to create an intracellular niche *in vivo*. This niche protects *H. pylori* from antibiotics and leads to infection recrudescence after therapy (10). We recently described an evasion strategy where *H. pylori* subverts autophagosomes into a pro-survival niche by disrupting lysosomal acidification and maturation, enabling the bacteria to survive within the host cell and establish persistent colonization (11, 12). However, to date, studies to systemically characterize how intracellular *H. pylori* reprograms host cell transcriptomes and how *cagA* contributes to colonization within the host cell are lacking.

The advent of dual RNA sequencing (RNA-seq) through probe-independent massively parallel cDNA sequencing now offers the opportunity for the comprehensive and simultaneous whole-genome transcriptional profiling of both the pathogen and infected host cells at high resolution (13–15). Using this technology, we infected a normal human gastric epithelial cell line (GES-1) with wild-type (WT) or *cagA*-mutated *H. pylori* strains to monitor the progression of *H. pylori* infection over time and simultaneously generated high-resolution transcriptome profiles of *H. pylori* and the human host cells. The depth of sequencing used allowed us to identify infection-specific host transcriptome alterations and characterize the adaption and pathogenesis strategies of the microorganism during infection. In addition, we performed *in vitro* and *in vivo* experiments to validate the findings obtained from the dual RNA-seq data set. Collectively, this study is the first to perform a dual-transcriptome analysis of *H. pylori* during dynamic interaction with the gastric epithelium and to provide new insights into the pathogenesis of *H. pylori*.

## RESULTS

### Identification of host-induced *H. pylori*-specific stress responses using dual RNA-seq

To explore the interaction between intracellular *H. pylori* and the human gastric epithelium, we first labeled *H. pylori* with fluorescein isothiocyanate (FITC) and determined the internalization efficiency of this microorganism by coculturing it with GES-1 cells for 3 or 24 h. We observed that most FITC-labeled *H. pylori* were stained by the antibody in Triton X-100-permeabilized GES-1 cells. However, in cells not treated with Triton X-100, these FITC-labeled bacteria could not be detected by the antibody (Fig. S1A), indicating that *H. pylori* can invade GES-1 cells as early as 3 h post coincubation. Gentamicin, an aminoglycoside antibiotic that poorly penetrates the eukaryotic cell membrane (16), was used to eliminate extracellular bacteria. We found that treatment with gentamicin (100 µg/mL) efficiently inhibited the biofilm formation (Fig. S1B) and bacterial growth (Fig. S1C) of multiple *H. pylori* strains, including TN2GF4, NCTC 11637, PMSS1, and 7.13. A recent study suggested that gentamicin exerts adverse effects, such as mitochondrial damage in cell culture (17). Thus, we examined whether gentamicin supplementation could induce reactive oxygen species and the associated DNA damage in GES-1 cells. Our results revealed that gentamicin at a concentration of 100 µg/mL had no adverse impact on the proliferation of GES-1 cell biofilm formation (Fig. S1D). Moreover, in contrast to carbonyl cyanide 3-chlorophenylhydrazone, we did not observe an increased production of mitochondrial superoxide in gentamicin-treated GES-1 cell biofilm formation (Fig. S1E). Similarly, the level of 8-hydroxy-2′-deoxyguanosine (8-OHdG), a hallmark of DNA damage, was not significantly increased in cells coincubated with gentamicin biofilm formation (Fig. S1E). These results collectively demonstrated that gentamicin at a concentration of 100 µg/mL effectively restricted the growth of *H. pylori* but had minimal adverse effects on the proliferation of GES-1 cells.

**Fig 1:**
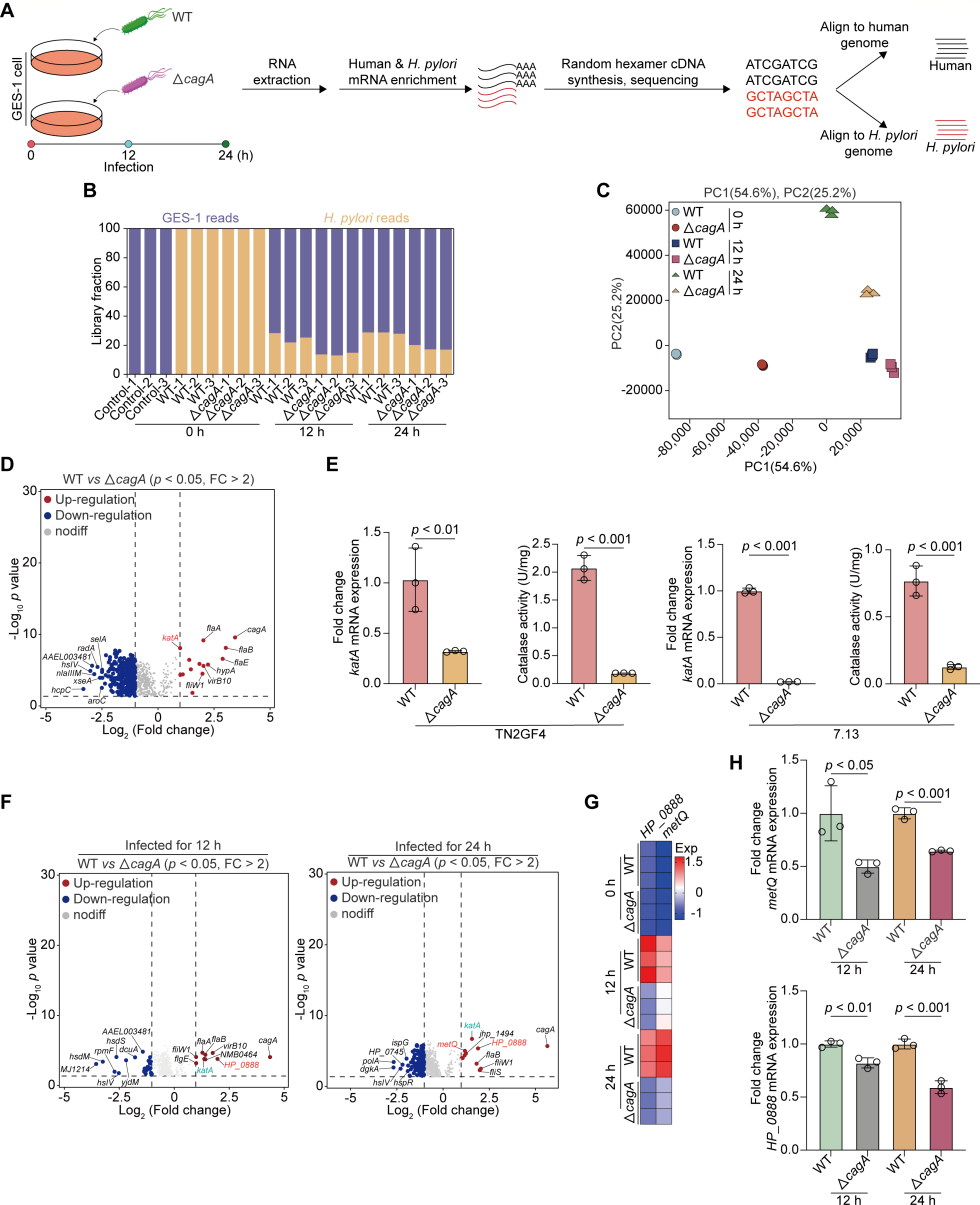
Identification of host-induced *H. pylori*-specific stress responses using dual RNA-Seq. (**A–D**) GES-1 cells were infected with WT or *cagA*-mutated *H. pylori* TN2GF4 strains (MOI 100) for 3 h and then exposed to gentamicin (100 µg/mL) for 12 or 24 h to eliminate the extracellular bacteria as detailed in “supplemental Materials and Methods.” (**A**) Experimental workflow for dual RNA-seq RNA extraction, library preparation, and sequencing processing. (**B**) The proportions of reads aligned to *H. pylori* or GES-1 cells in each library. On average, there are 46 million reads per library: 71.6% of the reads aligned to the human genome and 28.4% to the *H. pylori* genome. (**C**) Principal component analysis (PCA) of the *H. pylori* transcriptome from different experiment conditions. (**D**) Volcano plot showing the DEGs obtained from WT *H. pylori* TN2GF4 strain compared with *cagA*-mutated TN2GF4 strain at time 0 using the *DESEq2* toolkit. *P* value < 0.05 was considered statistically significant, Benjamini-Hochberg adjusted two-sided Wilcoxon test. (**E**) Total mRNA or proteins were extracted from WT or *cagA*-mutated *H. pylori* TN2GF4 (left) or 7.13 (right) strains. The expression levels of *katA* mRNA and catalase activity were determined. *H. pylori* 16S rRNA was used as the loading control for *katA* mRNA. (**F**) Volcano plot showing the DEGs obtained from WT *H. pylori* TN2GF4 strain compared with *cagA*-mutated TN2GF4 strain after coculturing with GES-1 cells for 12 (left) or 24 h (right). *P* value < 0.05 was considered statistically significant, Benjamini-Hochberg adjusted two-sided Wilcoxon test. (**G**) Heatmap showing the expression of ATP-binding cassette (ABC) transporter-related genes, *metQ* and *HP_0888*, in WT or *cagA*-mutated *H. pylori* TN2GF4 strains after coculturing with GES-1 cells for 0, 12, or 24 h. Color coding was based on normalized expression levels. (**H**) GES-1 cells were infected with WT or *cagA*-mutated *H. pylori* TN2GF4 strains (MOI 100) for 3 h and then exposed to gentamicin (100 µg/mL) for 12 or 24 h to eliminate the extracellular bacteria. The expression levels of *metQ* (upper) and *HP_0888* (down) mRNA were determined. *H. pylori* 16S rRNA was used as the loading control. All the quantitative data were presented as means ± SD from three independent experiments. *P < 0.05, **P < 0.01, and ***P < 0.001.

Next, we cocultured GES-1 cells with WT or △*cagA H. pylori* TN2GF4 strains at a multiplicity of infection (MOI) of 100 for 3 h. Subsequently, gentamicin (100 µg/mL) was used to eliminate extracellular bacteria. We monitored genome-scale transcriptomic events during the infection by performing dual RNA-seq on both infected and mock-infected GES-1 cells at time 0 (uninfected GES-1 cells and *H. pylori* before infection) and 12 and 24 h post-infection, with three biologically independent experiments per timepoint (Fig. 1A). The sequencing libraries yielded over 121 million total reads (median, 73,752,510 reads; range, 9,815,954 to 86,633,416 reads). After adapter trimming and removal of low-quality reads, we retained an average of 46 million clean reads per sample (median, 54,816,222 reads; range, 9,309,838 to 70,169,125 reads), of which approximately 28.4% and 71.6% of the total reads originated from the pathogen and the human genome, respectively (Fig. 1B). As anticipated, only 0.72% of human and 0.06% of *H. pylori* reads were mapped and counted as ribosomal RNAs, indicating the successful depletion of host and bacterial rRNAs. The high number of usable reads in each library indicated the reliability of our dual RNA-seq approach, including total RNA extraction, host and bacterial mRNA enrichment, and cDNA library preparation, which enabled us to determine different gene expression patterns in host-pathogen transcripts with high resolution.

We systematically profiled the transcriptional events of *H. pylori* at each timepoint to identify infection-specific gene expression, which may lead to adjustments in the virulence and fitness of the pathogen. PCA showed distinct global gene expression in WT and *cagA*-mutant *H. pylori* at different incubation periods, and the profiles of the three biological replicates were clustered together (Fig. 1C). To investigate the *cagA*-dependent transcriptome in *H. pylori*, we analyzed gene expression profiles in WT and △*cagA H. pylori* strains at time 0 using the differential expression analysis package *DESeq2* with a twofold cutoff and a Benjamini-Hochberg false discovery rate of <0.05 as the threshold for inclusion. We identified 14 genes with significantly higher expression in WT *H. pylori* than in the *cagA*-mutant strain (Fig. 1D). Moreover, the expression level of *cagA* was the highest in WT *H. pylori* [Fold change (FC) = 10.9, *P* = 3.0E^−10^], indicating the successful mutation of the *cagA* factor. A set of genes related to the flagellar regulatory system, including *flaB* (FC = 8.24, *P* = 8.6E^−09^), *flgE* (FC = 7.47, *P* = 2.9E^−07^), *flaA* (FC = 4.12, *P* = 7.7E^−10^), *fliW1* (FC = 4.00, *P* = 3.5E^−05^), *fliS* (FC = 2.93, *P* = 0.01), *fliD* (FC = 2.66, *P* = 4.1E^−07^), *motB* (FC = 2.17, *P* = 4.4E^−05^), and *motA* (FC = 2.02, *P* = 5.12E^−05^), was significantly downregulated in the *cagA* isogenic mutant strain. This finding is consistent with that of a previous study indicating that *cagA* is associated with bacterial motility (18). Moreover, we observed a marked reduction in the expression level of *katA* in the △*cagA* strain (FC = 2.0, *P* = 9.4E^−09^). The *katA* (catalase) gene of *H. pylori* encodes an antioxidant enzyme that protects the bacteria from oxidative stress (19). To verify whether the *cagA* mutation is responsible for the decreased expression of *katA*, we measured the *katA* mRNA level and catalase activity in two *H*. *pylori* strains, TN2GF4 and 7.13, with or without the *cagA* mutation. We observed that the levels of *katA* mRNA expression and catalase activity were both significantly decreased in △*cagA* strains (Fig. 1E), indicating that *katA* is transcriptionally inactivated in the absence of *cagA* expression in *H. pylori*. In contrast, we identified that a cluster of genes, including *hcpC* (Sel1 repeat family protein), *nlaIIIM* (DNA adenine methylase), and *radA* (DNA repair protein), were upregulated in the *cagA* isogenic mutant strain (Fig. 1D).

Next, we compared transcriptional diversity between WT and *cagA-*mutated strains after coculturing with GES-1 cells for 12 or 24 h. Our findings revealed that *katA* and flagellum-related genes were highly expressed in the transcriptome of the WT *H. pylori* strain (Fig. 1F). Moreover, compared with △*cagA* strains, *HP_0888* and *metQ* were both identified to be strongly upregulated in the WT *H. pylori* strain upon coincubation with GES-1 cells for 12 (*HP_0888*: FC = 4.0, *P* = 1.8E^−04^; *metQ*: FC = 1.3, *P* = 9.8E^−04^) or 24 h (*HP_0888*: FC = 3.8, *P* = 4.9E^−06^; *metQ*: FC = 2.3, *P* = 1.1E^−05^, Fig. 1F). *HP_0888* and *metQ* are both critical components of ABC transporters that mediate the uptake of host-provided nutrients into the cytoplasm of *H. pylori* for its proliferation and pathogenicity (20, 21). Heatmap analysis showed that such upregulation occurred after coculturing of *H. pylori* with GES-1 cells (Fig. 1G). Furthermore, we performed quantitative real-time PCR (qRT-PCR) and determined that the expression of *HP_0888* and *metQ* was both significantly declined in *cagA-*mutated strains after coculturing them with GES-1 cells for 12 or 24 h (Fig. 1H). This finding indicated that ABC transporter genes, *HP_0888* and *metQ*, were regulated by *cagA* in response to host-imposed stresses during *H. pylori* infection.

### *H. pylori cagA* activated oxidative phosphorylation by disrupting electron transportation

**Fig 2:**
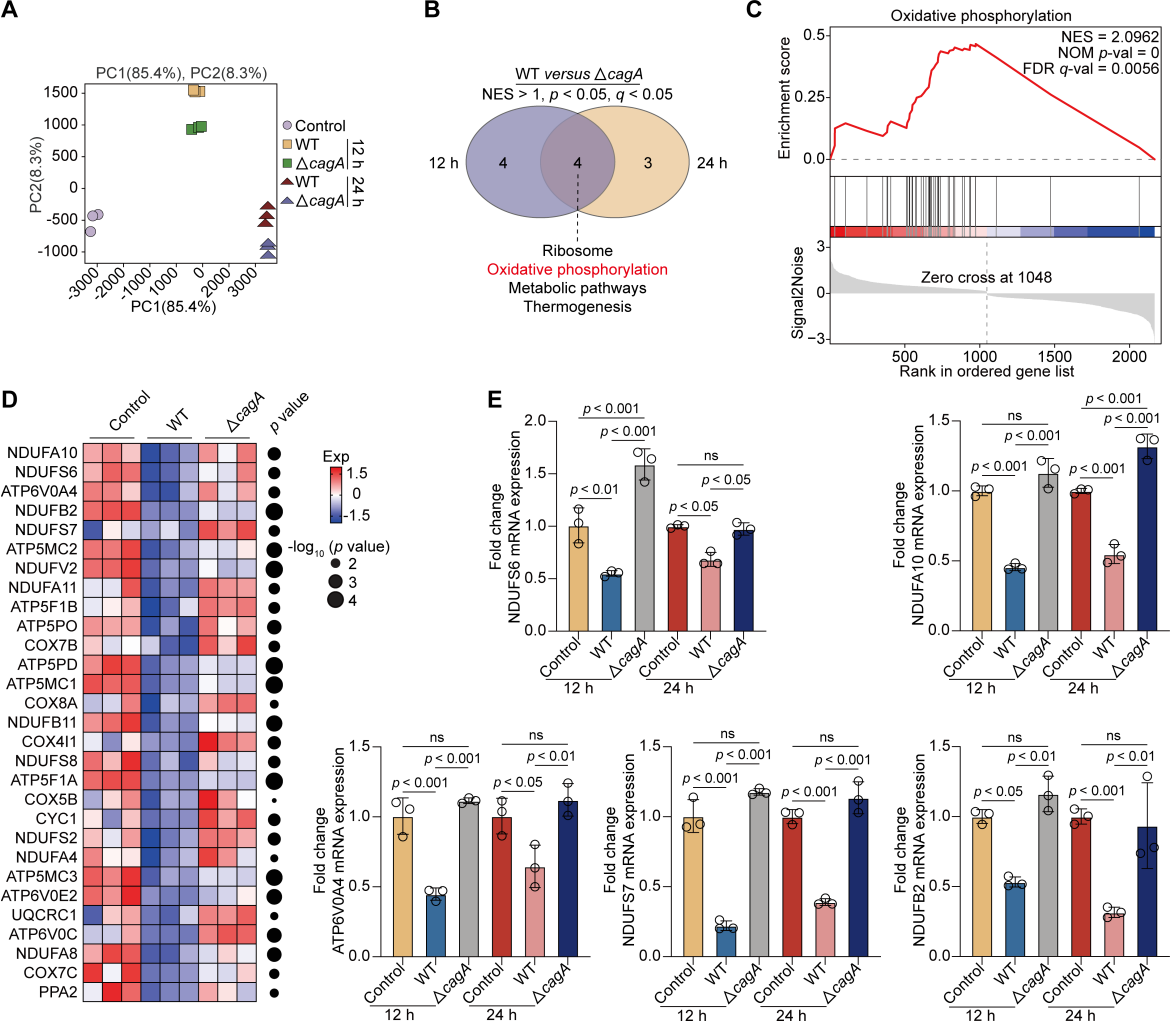
*H. pylori cagA* activated oxidative phosphorylation by disrupting the electron transportation. (**A–D**) GES-1 cells were infected with WT or *cagA*-mutated *H. pylori* TN2GF4 strains (MOI 100) for 3 h and then exposed to gentamicin (100 µg/mL) for 12 or 24 h to eliminate the extracellular bacteria. (**A**) PCA diagram showing the host cell transcriptome from different experiment conditions. (**B**) Venn diagram showing the number of GSEA-enriched biological pathways in GES-1 cells infected with WT versus *cagA*-mutated *H. pylori* TN2GF4 strains for 12 (left) or 24 h (right), with four pathways including oxidative phosphorylation (red) being simultaneously enriched in both two timepoints. (**C**) GSEA enrichment plot showed that the “oxidative phosphorylation” pathway was enriched in GES-1 cells infected with WT versus *cagA*-mutated *H. pylori* TN2GF4 strains for 24 h. (**D**) Heatmap showing the expression of ETC-related genes in uninfected GES-1 cells or cells infected with WT or *cagA*-mutated *H. pylori* TN2GF4 strains for 24 h. Dot size indicates the −log_10_ transformed *P* values, color coding based on normalized expression levels. (**E**) GES-1 cells were infected with WT or *cagA*-mutated *H. pylori* TN2GF4 strains (MOI 100) for 3 h and then exposed to gentamicin (100 µg/mL) for 12 or 24 h to eliminate the extracellular bacteria. The mRNA expression of *NDUFS6*, *NDUFA10*, *ATP6V0A4*, *NDUFS7*, and *NDUFB2* was determined. β-Actin was used as the loading control. All the quantitative data were presented as means ± SD from three independent experiments. *P < 0.05, **P < 0.01, and ***P < 0.001.

To explore *H. pylori cagA*-reprogrammed host transcriptomic profiles, we performed PCA analysis and observed a clear separation in GES-1 cells infected with WT or *cagA*-mutated *H. pylori* strains for 12 or 24 h (Fig. 2A). Next, using *DESeq2*, we identified 176 DEGs (FC > 2 or <0.5, *P* < 0.05) in GES-1 cells infected with the *H. pylori* WT strain compared with the *cagA*-mutated strain after 12 h post-challenge. Using the same filter criteria, we profiled 222 DEGs whose expression was either positively or negatively regulated by *H. pylori cagA* after 24 h of infection. Subsequently, we analyzed these DEGs to predict their biological functions through gene set enrichment analysis (GSEA) and identified four biological processes, including oxidative phosphorylation, common to both timepoints (Fig. 2B and C). Oxidative phosphorylation, a crucial source of ATP generation, supports cell growth and the progress of intracellular metabolic pathways (22, 23). This process is accomplished by transporting electrons to a series of transmembrane proteins located in the mitochondrial inner membrane, known as the electron transport chain (ETC) (24). Impaired electron transport leads to the aberrant accumulation of reactive oxygen species (ROS). We identified genes responsible for *cagA*-driven oxidative stress and observed a pronounced reduction in genes related to the ETC in GES-1 cells infected with the WT *H. pylori* strain compared with their control counterparts (Fig. 2D). These included genes encoding the subunits of NADH: ubiquinone oxidoreductase (*NDUFA10*, *NDUFS6*, *NDUFB2*, and *NDUFS7*), cytochrome *c* oxidase (*COX7B*, *COX8A*, *COX4I1*, *COX5B*, and *COX7C*), ATP synthase (*ATP6V0A4*, *ATP5MC2*, *ATP5F1B*, and *ATP5MC1*), *CYC1* (encoding cytochrome *c*), *PPA2*, and *UQCRC1*. However, this reduction was significantly reversed in △*cagA*-infected GES-1 cells. Furthermore, we performed qRT-PCR and verified that the mRNA expression of *NDUFS6*, *NDUFA10*, *ATP6V0A4*, *NDUFS7*, and *NDUFB2* was significantly decreased in GES-1 cells infected with the WT *H. pylori* strain for 12 or 24 h. Consistently, the reduction in the expression of these genes was significantly mitigated in GES-1 cells co-coculturing with the △*cagA H. pylori* strain (Fig. 2E), indicating that *cagA* triggered oxidative stress by disrupting electron transportation in GES-1 cells.

### Repressed expression of multiple splicing factors in response to *H. pylori* infection

**Fig 3:**
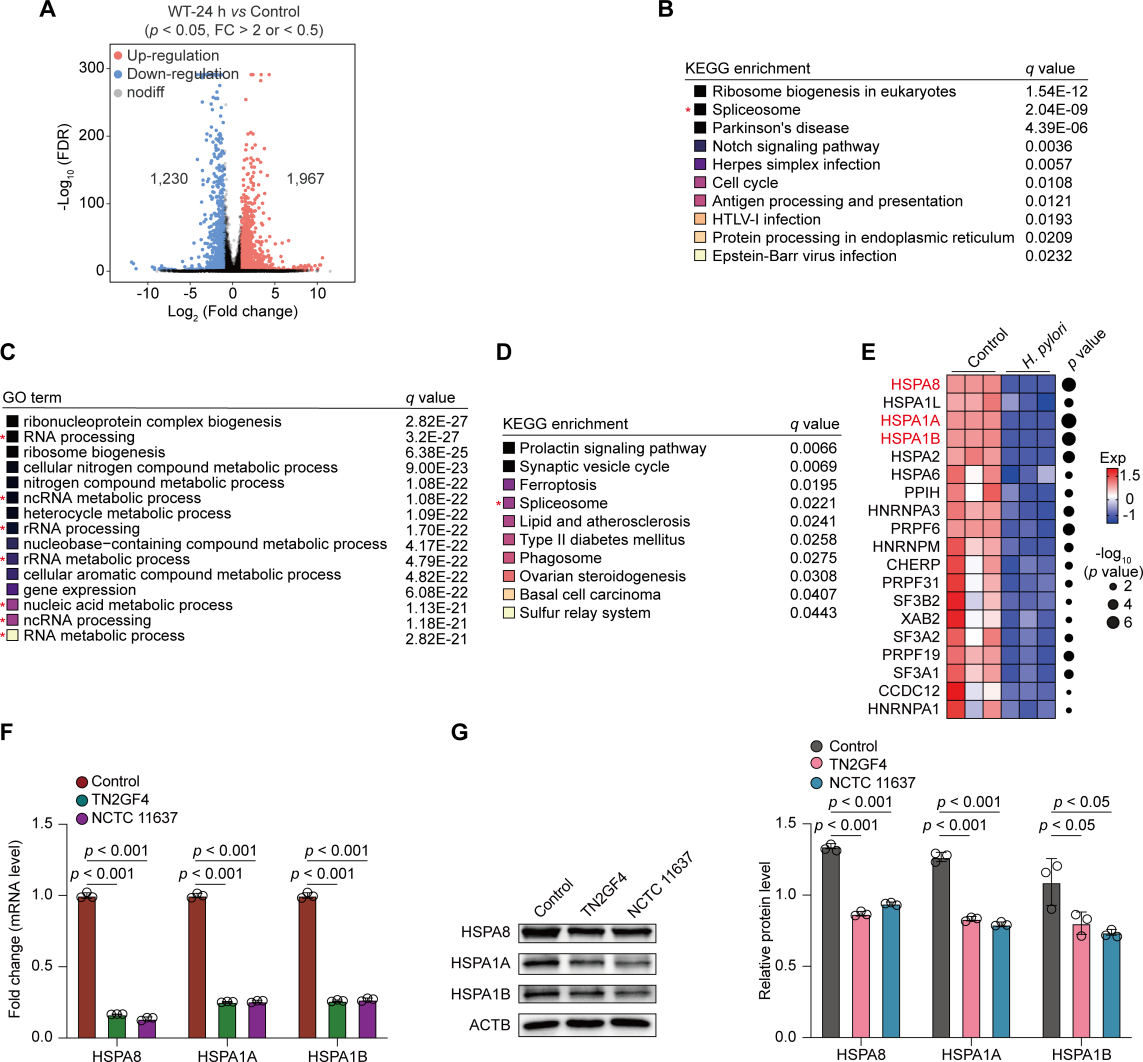
Repressed expression of multiple splicing factors in response to *H. pylori* infection. (**A–E**) GES-1 cells were infected with WT or *cagA*-mutated *H. pylori* TN2GF4 strains (MOI 100) for 3 h and then exposed to gentamicin (100 µg/mL) for 24 h to eliminate the extracellular bacteria. (**A**) Volcano plot showing the DEGs obtained from GES-1 cells infected with WT *H. pylori* TN2GF4 strain for 24 h compared with mock infection (FC > 2 or <0.5, *P* value < 0.05). Of these, 1,967 DEGs were upregulated, whereas 1,230 DEGs were downregulated in *H. pylori*-infected cells. Benjamini-Hochberg adjusted two-sided Wilcoxon test. (**B**) KEGG annotation of the top 10 enriched biological pathways using the downregulated DEGs from WT *H. pylori* TN2GF4-infected GES-1 cells compared with mock infection after 24 h post-challenge. Colored squares represented the *q* value (black, small; yellow, big). (**C**) GO annotation of the top 15 enriched biological pathways using the downregulated DEGs from WT *H. pylori* TN2GF4-infected GES-1 cells compared with mock infection after 24 h post-challenge. Colored squares represented the *q* value (black, small; yellow, big). (**D**) KEGG annotation of the top 10 enriched biological pathways using the downregulated DEGs from *cagA*-mutated *H. pylori* TN2GF4-infected GES-1 cells compared with mock infection after 24 h post-challenge. Colored squares represented the *q* value (black, small; yellow, big). (**E**) Heatmap showing the expression of splicing regulators in uninfected GES-1 cells or cells infected with WT *H. pylori* TN2GF4 strains for 24 h. Dot size indicates the −log_10_ transformed *P* values, color coding based on normalized expression levels. (**F and G**) GES-1 cells were infected with *H. pylori* TN2GF4 or NCTC11637 strains (MOI 100) for 3 h and then exposed to gentamicin (100 µg/mL) for 24 h to eliminate the extracellular bacteria. The mRNA (**F**) and protein (**G**) expression of *HSPA8*, *HSPA1A*, and *HSPA1B* was determined in uninfected GES-1 cells or cells infected with *H. pylori* TN2GF4 or NCTC 11637 strains. β-Actin was used as the loading control. All the quantitative data were presented as means ± SD from three independent experiments. *P < 0.05 and ***P < 0.001.

Next, we characterized *H. pylori*-driven transcriptomic events in GES-1 cells. To examine gene expression profiles correlated with *H. pylori* infection, we used the *DESeq2* toolkit and identified 3,197 DEGs in GES-1 cells infected with *H. pylori* for 24 h compared with mock infection (FC > 2 or <0.5, *P* < 0.05). Of these, 1,967 genes were upregulated and 1,230 genes were downregulated in *H. pylori*-infected cells (Fig. 3A), suggesting altered transcriptional activity in response to *H. pylori* infection. Kyoto Encyclopedia of Genes and Genomes (KEGG) analysis revealed that DEGs downregulated in response to *H. pylori* infection were enriched in the spliceosome pathway (Fig. 3B). To further confirm the KEGG annotation results, we performed Gene Ontology (GO) analysis and determined that these downregulated genes were enriched in RNA/rRNA/ncRNA processing and ncRNA/rRNA/nucleic acid metabolic processes (Fig. 3C). Using the same filter criteria, we profiled 2,003 DEGs that were downregulated in △*cagA*-infected GES-1 cells compared with mock-infected cells (FC < 0.5, *P* < 0.05). KEGG analysis revealed that the DEGs downregulated in response to △*cagA H. pylori* strain infection were also enriched in the spliceosome pathway (Fig. 3D), suggesting that *H. pylori*-perturbed RNA splicing and RNA stability processes were not dependent on *cagA* expression.

We explored the transcriptional profile of splicing factors involved in the spliceosome pathway and identified that numerous well-established splicing regulators were downregulated in response to *H. pylori* infection, including heat shock proteins (*HSPs: HSPA8*, FC = 0.069, *P* = 1.84E^−07^; *HSPA1L*, FC = 0.302, *P* = 0.0028; *HSPA1A*, FC = 0.082, *P* = 1.51E^−08^; *HSPA1B*, FC = 0.115, *P* = 4.49E^−07^; *HSPA2*, FC = 0.278, *P* = 1.94E^−05^; and *HSPA6*, FC = 0.250, *P* = 0.018), splicing factor 3a Subunit 2 (*SF3A2*, FC = 0.599, *P* = 0.0066), splicing factor 3b subunit 2 (*SF3B2*, FC = 0.684, *P* = 0.043), heterogeneous nuclear ribonucleoprotein M (*HNRNPM*, FC = 0.44, *P* = 0.002), and heterogeneous nuclear ribonucleoprotein A3 (*HNRNPA3*, FC = 0.429, *P* = 2.6E^−03^) and heterogeneous nuclear ribonucleoprotein A1 (*HNRNPA1*, FC = 0.766, *P* = 0.04, Fig. 3E). Consistent with the dual RNA-seq data, coculturing GES-1 cells with two *H. pylori* strains, TN2GF4 and NCTC 11637, revealed the downregulation of *HSPA8*, *HSPA1A*, and *HSPA1B* at both mRNA and protein levels compared with mock treatment (Fig. 3F and G), indicating that *H. pylori* disrupted RNA splicing and RNA stability by reducing the expression levels of several splicing factors in GES-1 cells.

### *H. pylori* infection modulates mRNA splicing of functional genes involved in the cell cycle process

**Fig 4:**
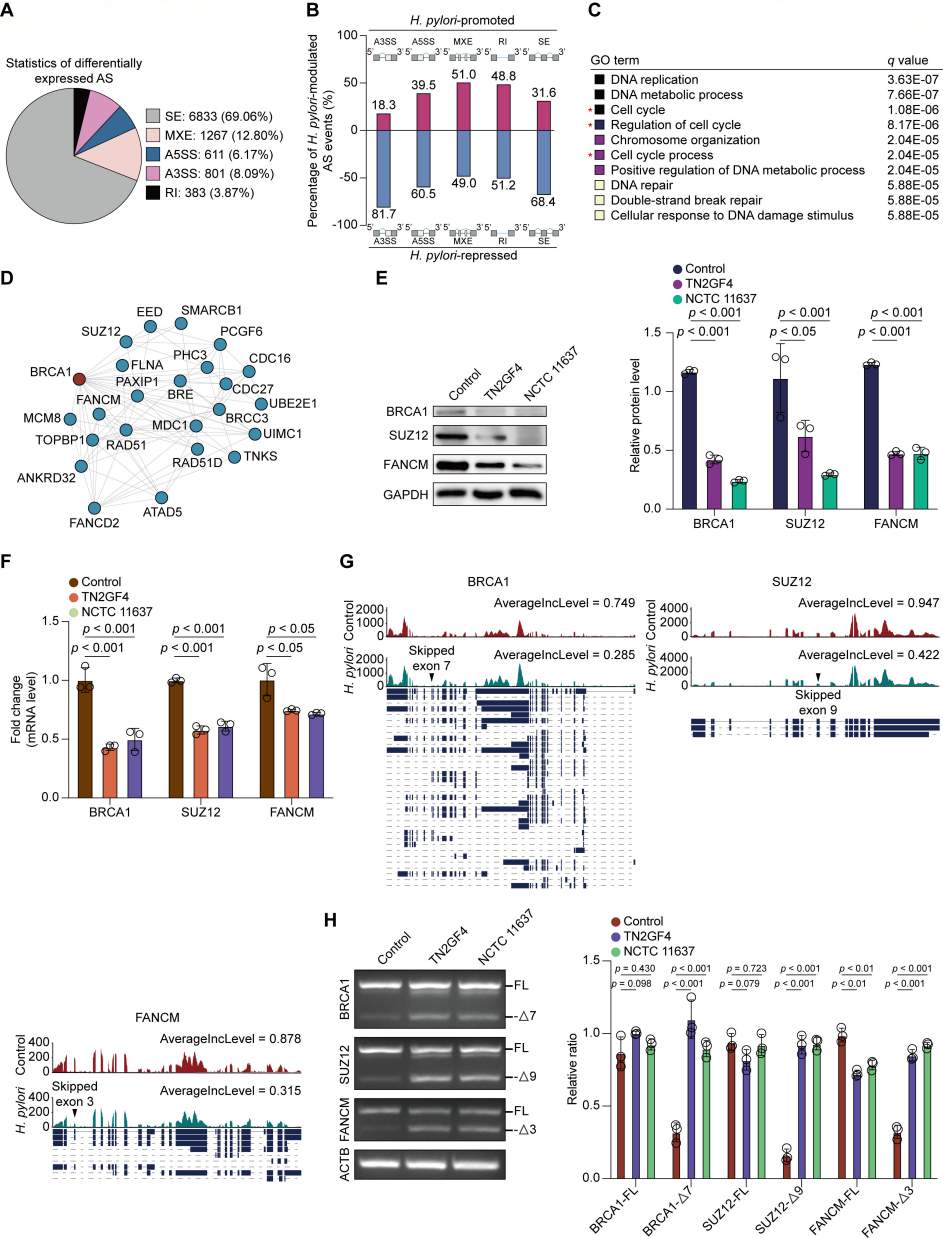
*H. pylori* infection modulated mRNA splicing of functional genes that were involved in cell cycle process. (**A–D**) GES-1 cells were infected with WT *H. pylori* TN2GF4 strain (MOI 100) for 3 h and then exposed to gentamicin (100 µg/mL) for 24 h to eliminate the extracellular bacteria. (**A**) Pie charts showing the proportion of each type of significantly altered splicing events in *H. pylori*-infected GES-1 cells compared with the controls using the rMATS algorithm. (**B**) Percentage of *H. pylori*-promoted or repressed splicing events in GES-1 cells. (**C**) GO annotation of the top 10 enriched biological pathways using the functional genes affected by *H. pylori*-promoted splicing events. Colored squares represented the *q* value (black, small; yellow, big). (**D**) The protein-protein interaction network for functional genes involved in the cell cycle process based on the STRING database. (**E and F**) GES-1 cells were infected with *H. pylori* TN2GF4 or NCTC11637 strains (MOI 100) for 3 h and then exposed to gentamicin (100 µg/mL) for 24 h to eliminate the extracellular bacteria. (**E**) The protein levels of BRCA1, SUZ12, and FANCM were determined and quantified in uninfected GES-1 cells or cells infected with *H. pylori* TN2GF4 or NCTC 11637 strains for 24 h. GAPDH was used as the internal control. (**F**) The mRNA expression of *BRCA1*, *SUZ12*, and *FANCM* was determined in uninfected GES-1 cells or cells infected with *H. pylori* TN2GF4 or NCTC 11637 strains for 24 h. (**G**) Exon skipping in the seventh exon of *BRCA1*, the nineth exon of *SUZ12*, and the third exon of *FANCM* as visualized by the IGV software. Black arrowheads indicate splicing sites. (**H**) GES-1 cells were infected with *H. pylori* TN2GF4 or NCTC11637 strains (MOI 100) for 3 h and then exposed to gentamicin (100 µg/mL) for 24 h to eliminate the extracellular bacteria. RT-PCR analysis of alternative splicing patterns of the changed splicing genes in control and *H. pylori*-infected GES-1 cells. β-Actin was used as the internal control. The expression of full-length and exon-skipping isoforms of the three genes was quantified. All the quantitative data were presented as means ± SD from three independent experiments. *P < 0.05, **P < 0.01, and ***P < 0.001.

Because the DEGs were enriched in the spliceosome pathway, we quantified splicing changes caused by *H. pylori* infection in GES-1 cells using the rMATS algorithm (25). Five major modes of alternative splicing (AS) events, namely, alternative 5′ splice sites (A5SS), alternative 3′ splice sites (A3SS), mutually exclusive exons (MXE), retained intron (RI), and skipped exons (SE), have been described in metazoan organisms (26). Using an adjusted *P* value cutoff of <0.05, we identified 9,895 splicing events that significantly changed in *H. pylori*-infected GES-1 cells compared with the controls (Fig. 4A). Specifically, the majority of AS events were exon skipping (69.06%), with 3,062 of the remaining events categorized into other splicing modes, including 1,267 for MXE, 611 for A5SS, 801 for A3SS, and 383 for RI. Thus, these data support the involvement of *H. pylori* in mRNA splicing during coculturing with GES-1 cells, with cassette exons being the most frequent targets upon *H. pylori* challenge.

To gain deep insights into the spectrum of genes that were aberrantly spliced, we defined *H. pylori*-modulated AS events using a stringent filter criteria (IncLevelDiff ≥ 0.1 or ≤−0.1, adjusted *P* < 0.05, and splice junction read coverage ≥ 20). We identified 736 *H. pylori*-promoted and 1,294 *H. pylori*-repressed splicing events in 628 and 1,064 genes in GES-1 cells, respectively (Fig. 4B). Approximately 65.8% of *H. pylori-*modulated AS events was SE, followed by MXE (15.2%), A3SS (8.7%), A5SS (6.1%), and RI (4.2%). Next, we focused on the 628 genes affected by *H. pylori*-promoted AS events and predicted their molecular functions by performing GO term enrichment analysis. We determined that 106 of these genes, including breast cancer type 1 (*BRCA1*), SUZ12 polycomb repressive complex 2 subunit (*SUZ12*), and FA complementation group M (*FANCM*), were involved in the cell cycle process (Fig. 4C). The protein-protein interaction network constructed using the STRING database further confirmed extensive interactions among these genes (Fig. 4D). Subsequently, we validated the expression of *BRCA1*, *SUZ12*, and *FANCM* in *H. pylori*-infected GES-1 cells and observed significant abrogation of the mRNA and protein expression of these three genes in cells treated with *H. pylori* strains TN2GF4 and NCTC 11637 (Fig. 4E and F). The results of rMATS analysis revealed that *H. pylori* infection promoted the skipping of the seventh exon in *BRCA1*, the ninth exon in *SUZ12*, and the third exon in *FANCM*, as visualized using the Integrative Genomics Viewer program (Fig. 4G). To verify the aberrant splicing of these three genes, we performed semi-quantitative and real-time PCR experiments using specific primers for target genes. We observed that although the pre-mRNA levels of these three genes remained stable, the expression of the exon-skipping isoforms of *BRCA1* (exon 7), *SUZ12* (exon 9), and *FANCM* (exon 3) was significantly elevated upon *H. pylori* infection (Fig. 4H), indicating that *H. pylori* treatment led to aberrant pre-mRNA splicing of functional genes involved in the cell cycle process.

### Gastric *H. pylori* colonization reduced the severity of chronic DSS-induced colitis

**Fig 5:**
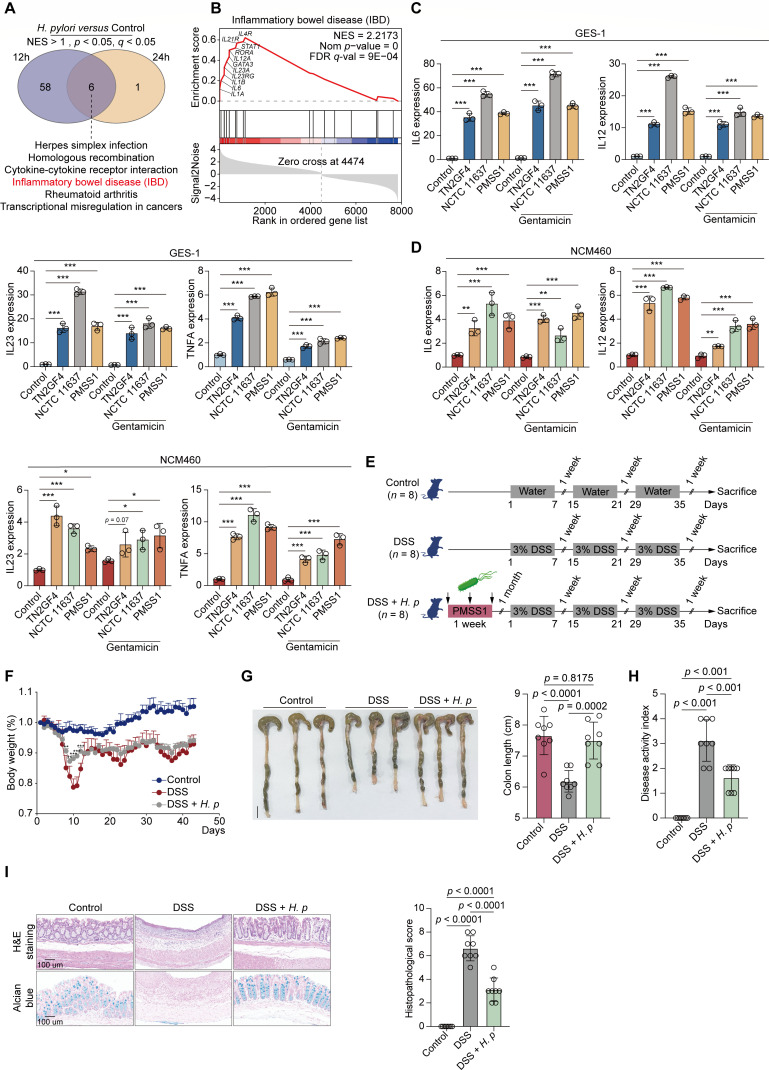
Gastric *H. pylori* colonization alleviated the severity of chronic DSS-induced colitis. (**A and B**) GES-1 cells were infected with WT *H. pylori* TN2GF4 strain (MOI 100) for 3 h and then exposed to gentamicin (100 µg/mL) for 12 or 24 h to eliminate the extracellular bacteria. (**A**) Venn diagram showing the number of GSEA-enriched biological pathways in *H. pylori*-infected GES-1 cells compared with mock infection after 12 or 24 h challenge, with six pathways including IBD pathway (red) being simultaneously enriched in both two timepoints. (**B**) GSEA enrichment plot showed that the “IBD” pathway was enriched in *H. pylori*-infected GES-1 cells for 24 h compared with mock infection. IBD pathway-related genes that were upregulated in response to *H. pylori* infection were labeled. (**C and D**) GES-1 (**C**) or NCM460 (**D**) cells were infected with *H. pylori* TN2GF4, NCTC 11637, or PMSS1 strains (MOI 100) for 24 h with or without gentamicin (100 µg/mL) treatment. The mRNA expression of *IL6*, *IL12*, *IL23*, and *TNFA* were determined. β-Actin was used as the loading control. The quantitative data were presented as means ± SD from three independent experiments. (**E–J**) C57BL/6J mice were orally inoculated with *H. pylori* PMSS1 strain (*n* = 8 animals) or the vehicle (*n* = 8 animals) for 1 month, followed by the administration of three cycles of 3% DSS (7 days/cycle), each separated by 7 days of regular water. (**E**) Schematic overview of the experimental design. (**F**) The changes of mice body weight after DSS administration were monitored. Mean ± SD from eight mice in each group. (**G**) (Left) Representative photographs of mouse colon tissue from each group were presented. Scale bar = 1 cm. (Right) The colon length of each mice was recorded. (**H**) The DAI index per mice was evaluated. (**I**) The histological analysis of mice colon tissue was performed by hematoxylin and eosin (H&E) and alcian blue staining. Scale bar = 100 µm. Histological scores of the DSS-induced colitis were evaluated. The quantitative data were presented as means ± SD. *P < 0.05, **P < 0.01, and ***P < 0.001.

As mentioned above, we identified 3,197 DEGs in GES-1 cells infected with *H. pylori* for 24 h compared with mock-infected cells (FC > 2 or <0.5, *P* < 0.05). To further explore infection-specific transcriptome signatures, we profiled 1,831 DEGs in *H. pylori*-infected GES-1 cells at 12 h using the same filter criteria. Of these, 808 upregulated and 1,023 downregulated DEGs were identified in *H. pylori*-infected GES-1 cells compared with the controls. We then performed GSEA analysis to examine biological functions in *H. pylori*-infected GES-1 cells at 12 or 24 h post-challenge by importing these DEGs. We observed that a cluster of inflammatory bowel disease (IBD)-related cytokines and genes was strongly upregulated in GES-1 cells infected with *H. pylori* for 12 or 24 h (Fig. 5A and B). Consistent with the dual RNA-seq data, coculturing of GES-1 cells with three *H. pylori* strains, namely, TN2GF4, NCTC 11637, and PMSS1, resulted in significantly elevated expression of IBD-associated genes, including *IL6*, *IL12*, *IL23*, and *TNFA*, upon *H. pylori* infection, regardless of whether eliminating extracellular pathogens by gentamicin or not (Fig. 5C).

Previous epidemiological data have indicated an inverse association between *H. pylori* infection and the risk of IBD due to the ability of *H. pylori* to induce immune tolerance (27). However, detailed mechanisms through which *H. pylori* infection protects against IBD still remain unclear. To assess whether *H. pylori* infection can stimulate a similar IBD-associated immune response in a normal human intestinal epithelial cell line, we selected NCM460 to be cocultured with these *H. pylori* strains, such as TN2GF4, NCTC 11637, and PMSS1, with or without gentamicin administration. Consistently, we discovered that *H. pylori* coincubation substantially elevated the expression of *IL6*, *IL12*, *IL23*, and *TNFA*, regardless of the use of gentamicin to eliminate extracellular pathogens (Fig. 5D).

We further investigated whether *H. pylori* infection can efficiently protect against the development of IBD in an *in vivo* model. We used the mouse-adapted, *cag*PAI-positive *H. pylori* strain PMSS1 to colonize the stomach of mice and then assessed whether gastric *H. pylori* colonization can mitigate the severity of chronic colitis induced by 3.0% dextran sulfate sodium (DSS) (Fig. 5E). We observed severe clinical symptoms in all colitis mice that received DSS, including loss of body weight and stool consistency, and stool with occult blood. However, mice precolonized with *H. pylori* exhibited significantly less body weight than those treated with DSS alone (Fig. 5F). Likewise, *H. pylori* pretreatment substantially alleviated the shortening of colon length in DSS-treated mice (Fig. 5G). Disease activity index (DAI) scores were markedly decreased in mice pretreated with *H. pylori* compared with those treated with DSS alone (Fig. 5H). Moreover, we collected colon tissues for histological assessment by performing H&E and alcian blue staining and observed weakened intestinal epithelial destruction, limited inflammatory cell infiltration, and declined goblet cell loss in *H. pylori* precolonized mice compared with those administered DSS alone (Fig. 5I). Thus, these findings collectively demonstrated that gastric *H. pylori* colonization significantly upregulated the expression levels of IBD-associated genes, efficiently attenuated colonic inflammation, and mitigated the destruction of the colonic mucosal structure caused by DSS administration.

### *H. pylori*-sustained *Muribaculaceae* abundance contributed to the restoration of intestinal barrier function damaged by DSS treatment

**Fig 6:**
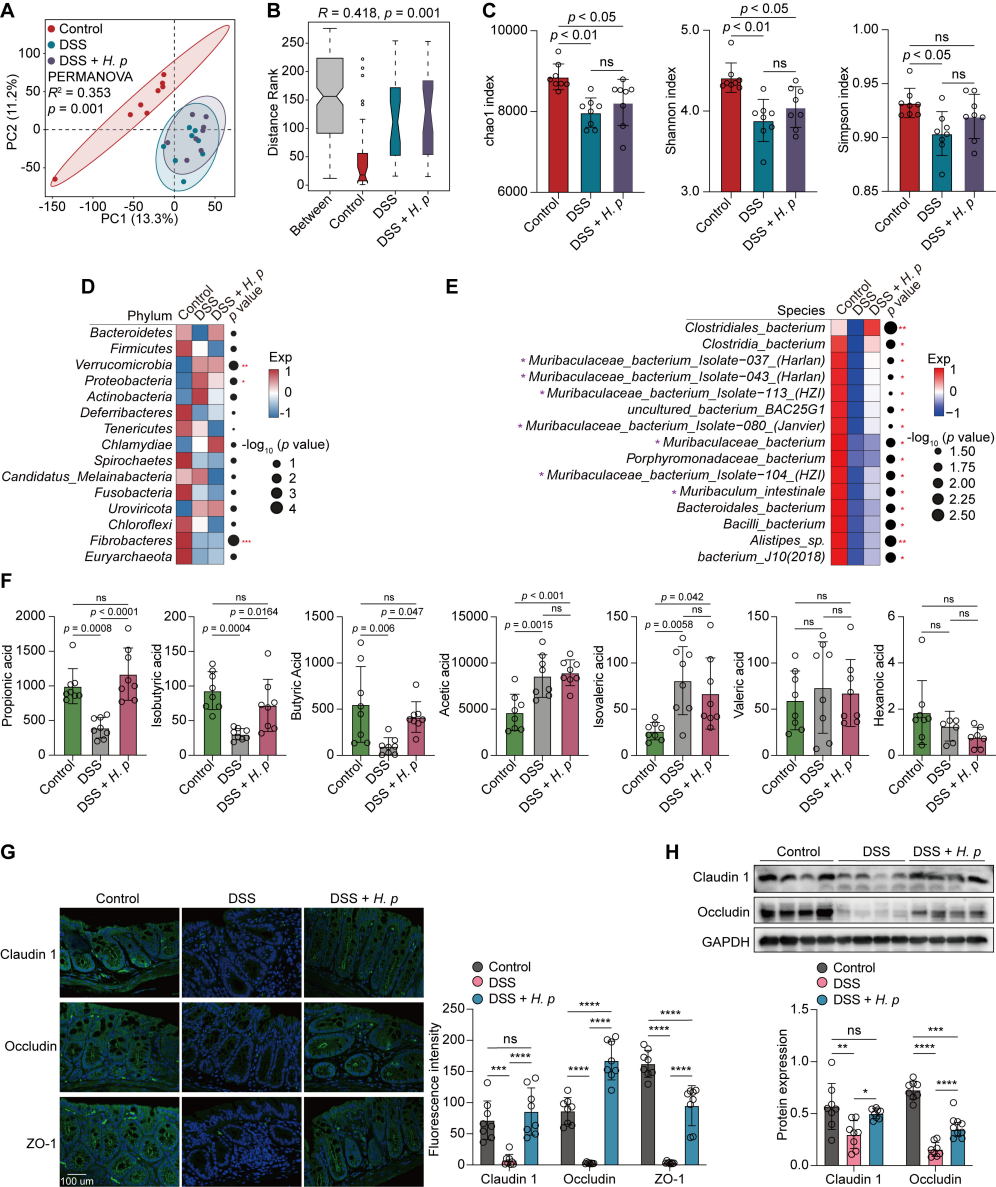
*H. pylori*-sustained *Muribaculaceae* abundance contributed to the restoration of intestinal barrier function damaged by DSS treatment. (**A–H**) C57BL/6J mice were orally inoculated with *H. pylori* PMSS1 strain (*n* = 8 animals) or the vehicle (*n* = 8 animals) for 1 month, followed by the administration of three cycles of 3% DSS (7 days/cycle), each separated by 7 days of regular water. (**A**) PCA diagram showing the β-diversity of mice fecal microbiota among the three groups at the species level. (**B**) ANOSIM test was applied to compare microbial structure dissimilarity between and within groups. Two-sided Wilcoxon rank-sum test. (**C**) The α-diversity of intestinal microbiota in the three groups at the species level was evaluated by chao1 (left), Shannon (middle), and Simpson (right) indices. (**D and E**) Analysis of the differences in the mice intestinal microbiota at the phylum (**D**) or species (**E**) levels. Dot size indicates the −log_10_ transformed *P* values, color coding based on normalized expression levels. Two-sided Wilcoxon rank-sum test. (**F**) The concentrations of seven types of short-chain fatty acid (SCFA) in mice colon tissue from each group were determined by absolute quantitative metabolomics. (**G**) The fluorescence intensities of Claudin 1, Occludin, and ZO-1 in the mice colon were determined by immunofluorescence staining. Scale bar = 100 µm. (**H**) The protein levels of Claudin 1 and Occludin in the mice colon were determined and quantified by western blotting. GAPDH was used as the internal control. All the quantitative data were presented as means ± SD. *P < 0.05, **P < 0.01, and ***P < 0.001.

Disordered intestinal microbiota is the underlying pathogenic mechanism of IBD (28–30). To investigate whether intestinal flora is involved in *H. pylori*-alleviated colitis, we collected stool samples from these mice and subjected them to high-throughput shotgun metagenomic sequencing to profile their microbial community taxonomic composition. PCA at the species level revealed distinct structures of the intestinal microbiota among these three groups [*R*^2^ = 0.353, *P* = 0.001, permutational multivariate analysis of variance (PERMANOVA)] despite no marked structural changes between DSS alone and DSS and *H. pylori* cotreatment groups (Fig. 6A). This distinction was associated with differences in community composition, as determined by analysis of similarities (ANOSIM) analysis (*R* = 0.418, *P* = 0.001; Fig. 6B). This finding indicated a more heterogeneous community structure in DSS-treated and DSS and *H. pylori*-cotreated mice than in control mice. We further observed a decline in the α-diversity of the intestinal microbiota, as indicated by the chao1, Shannon, and Simpson indexes, in DSS-treated mice compared with their control counterparts. However, higher chao1, Shannon, and Simpson indexes were observed in *H. pylori*-pretreated mice compared with those treated with DSS alone, although the difference was not statistically significant (Fig. 6C). This finding suggested the recovery of the richness and diversity of the intestinal microbiota in *H. pylori*-precolonized mice.

Next, we performed metagenomic phylogenetic analysis (MetaPhlAn2) to examine taxonomic abundance in these fecal samples. We determined that at the phylum level, the abundance of *Bacteroidetes* was decreased in DSS-treated mice relative to the control mice, whereas its level was restored in *H. pylori*-pretreated mice (Fig. 6D). Conversely, the abundance of *Proteobacteria* was increased following DSS treatment, whereas *H. pylori* precolonization markedly limited its accumulation in the mouse colon (Fig. 6D**)**. At the species level, we identified that the abundance of a group of bacteria belonging to the *Muribaculaceae* family within the *Bacteroidetes* phylum was significantly decreased in DSS-treated mice, whereas their abundance was markedly sustained after *H. pylori* precolonization (Fig. 6E). These microorganisms included *Muribaculaceae_bacterium_Isolate−037_(Harlan)* (*P* = 0.03), *Muribaculaceae_bacterium_Isolate−043_(Harlan)* (*P* = 0.04), *Muribaculaceae_bacterium_Isolate−113_(HZI)* (*P* = 0.04), *Muribaculaceae_bacterium_Isolate−080_(Janvier)* (*P* = 0.04), *Muribaculaceae_bacterium* (*P* = 0.02), *Muribaculaceae_bacterium_Isolate−104_(HZI)* (*P* = 0.02), and *Muribaculum_intestinale* (*P* = 0.02).

*Muribaculaceae* has been newly identified as a type of SCFA-producing bacteria (31), especially propionate and butyrate (32). Their abundance was markedly decreased in mice with fatty liver disease (33). To determine whether the restored abundance of *Muribaculaceae* was accompanied by increased SCFA levels in *H. pylori*-pretreated mouse colon, we conducted absolute quantitative metabolomic analysis to measure SCFA levels in mouse stool samples. As expected, we discovered significant reductions in propionic acid, isobutyric acid, and butyric acid levels in DSS-treated mice, whereas the levels of these three SCFAs were substantially restored upon gastric *H. pylori* colonization (Fig. 6F), indicating that *H. pylori*-elevated *Muribaculaceae* bacteria may contribute to the enrichment of propionic and butyric acids in the mouse colon. However, this phenomenon was not observed for other SCFA members (Fig. 6F), suggesting that *Muribaculaceae* bacteria were preferentially responsible for the production of propionic and butyric acids but not the other SCFAs.

SCFAs, including propionate and butyrate, produced by bacterial fermentation in the gut, have long been of interest to researchers for their capacity to maintain intestinal barrier integrity and homeostasis (34). To explore whether *H. pylori* precolonization could effectively repair the intestinal barrier damaged by DSS, we determined the expression levels of three tight junction proteins, namely, Claudin 1, Occludin, and ZO-1, which are directly responsible for intestinal barrier function (35). As shown in Fig. 6G, the immunofluorescence intensity of these three proteins was significantly decreased in the colon of DSS-treated mice relative to those from the control counterparts. However, such reduction was markedly reversed by *H. pylori* precolonization. Consistently, immunoblotting analysis confirmed that the decreased protein levels of Claudin 1 and Occludin in DSS-treated mice were significantly restored by *H. pylori* pretreatment (Fig. 6H), indicating that the intestinal barrier integrity damaged by DSS treatment was recovered upon *H. pylori* colonization.

## DISCUSSION

The recent development of dual RNA-seq technology has enabled researchers to simultaneously profile RNA expression in host-pathogen systems, offering novel insights into complex host-pathogen interactions. In this study, by leveraging the advantages of this technology, we presented the first dual-transcriptome analysis of *H. pylori* and its human host, revealing the bacterium’s adaptation and pathogenesis mechanisms regulated by *cagA* during *H. pylori* infection of human gastric epithelial cells and exploring reprogrammed host transcriptomes in response to *H. pylori* colonization. The key events occurring during the interaction between *H. pylori* and host cells were further validated by performing *in vitro* and *in vivo* experiments using multiple standard bacterial strains.

*H. pylori* has a limited ability to use carbohydrates as a carbon source, forcing it to rely on exogenous amino acids and peptides provided by the host. The ABC transporter that mediates the import of these substrates into the cytoplasm of *H. pylori* is pivotal for bacterial pathogenicity and virulence (20). In this study, we discovered significantly elevated expression of ABC transporter-related genes, *metQ* and *HP_0888*, upon coculturing *H. pylori* with gastric epithelial cells. However, such upregulation was largely mitigated in the *cagA*-mutated strain, which suggests that *cagA* benefits intracellular *H. pylori* colonization by facilitating the uptake of host-provided nutrients. Moreover, we observed an inhibitory impact of *H. pylori cagA* on electron transportation, as evidenced by the general repression of a set of ETC-related genes. The disrupted oxidative phosphorylation process could lead to the accumulation of ROS in host cells, thereby causing DNA damage and chromosomal instability and thus offering a pathological potential for gastric carcinogenesis (36). These results enhance our understanding of the pathogenic mechanisms of *cagA*-positive *H. pylori* strains upon colonization in the human stomach.

As a fundamental mechanism responsible for protein polymorphism, AS is a central mode of genetic regulation in metazoan organisms (37). Previous studies have indicated a correlation between aberrant splicing and gastric diseases, especially adenocarcinoma (38, 39). Liu et al. recently analyzed AS events in *H. pylori*-negative gastric cancer patients using the TCGA data set and identified several survival-related AS events (40). However, a systematic profiling of AS events in response to *H. pylori* infection has yet to be conducted. In this study, we observed decreased expression of multiple splicing regulators, including *HSPAs*, *HNRNPs*, *SF3B2*, *SF3A1*, and *SF3A2*, in *H. pylori*-infected gastric epithelial cells. We globally analyzed *H. pylori*-regulated AS events using the rMATS algorithm, revealing that all common modes of AS occurred in *H. pylori*-infected GES-1 cells, with cassette exons being the most frequently targeted upon bacterial challenge. By profiling genes affected by *H. pylori*-promoted splicing events, we found that a substantial number of these genes were involved in the cell cycle process, which may partially explain the cell cycle arrest induced by *H. pylori* infection as reported in previous studies (41). Future studies are needed to determine whether *H. pylori*-induced aberrant splicing events are involved in the pathogenesis of this microorganism.

Numerous epidemiological studies have suggested a protective role for chronic *H. pylori* infection against IBD, with a lower *H. pylori* infection rate observed in patients with ulcerative colitis and Crohn’s disease than in those without IBD (42–44). Some researchers have attributed this protective effect to *H. pylori*-induced systemic immune tolerance or to medical therapies used by patients with IBD, including metronidazole (45–47). However, whether *H. pylori*-modulated immune response can affect the pathogenesis of IBD still remains debatable. Consistent with previous data, our results indicated that gastric *H. pylori* colonization significantly mitigated the severity of DSS-induced colitis, as evidenced by alleviated clinical symptoms in DSS-treated mice. Mechanistically, we are inspired by a recent review (30) and started to investigate whether *H. pylori*-altered intestinal microbiota was involved in protecting against DSS-induced colitis. Resultingly, we identified a cluster of propionic and butyric acid-producing bacteria, *Muribaculaceae*, selectively enriched in the colon of *H. pylori*-precolonized mice, which may contribute to the restoration of intestinal barrier function damaged by DSS treatment. Increased production of SCFAs is considered beneficial for health because these metabolites play a versatile role in supporting gut homeostasis (48). Thus, apart from *H. pylori*-induced immune tolerance, our findings indicate the potential involvement of the intestinal microbiota remodeled by *H. pylori* in protecting against IBD. Further elucidation of the mechanism underlying this protective effect can help in the development of effective strategies for the treatment and prevention of IBD.

In summary, this study presents a robust and systematic characterization of the interplay between *H. pylori* and human gastric epithelial cells, offering mechanistic insights into the bacterium’s adaptive and pathogenic strategies. These results enhance our understanding of how *H. pylori* promotes infection and pathogenesis in the human gastric epithelium and provide evidence to identify targets for antimicrobial therapies.

## MATERIALS AND METHODS

### Bacterial and cell culture

Wild-type and *cagA*-mutant *H. pylori* TN2GF4 strains, NCTC 11637 (ATCC43504), were obtained from the Department of Microbiology, The Chinese University of Hong Kong. The *H. pylori* strain PMSS1 and 7.13 wild-type and *cagA*-mutant strains were kind gifts from Dr. Chuan XIE (The First Affiliated Hospital of Nanchang University, Jiangxi, China). *H. pylori* was initially grown on horse blood agar plates (Columbia Blood Agar Base with DENT Selective Supplements by Oxoid) in an anaerobic jar with a microaerophilic environment for 5 days at 37°C. GES-1 (normal human gastric epithelial cell line) and NCM460 (normal human colon mucosal epithelial cell line) were from Dr. Wang Hong Ying (Chinese Academy of Medical Sciences, Beijing, China). GES-1 and NCM460 cells were cultured in Dulbecco’s modified Eagle’s medium (Gibco, Thermo Fisher) supplemented with 10% fetal bovine serum (Gibco, Thermo Fisher) at 37°C in 5% CO_2_.

### Reagents, antibodies, and commercial kits

Primary antibodies we used were anti-*H. pylori* (ab7788, Abcam), anti-HSPA8 (10654-1-AP, Proteintech), anti-HSPA1A (10995-1-AP, Proteintech), anti-HSPA1B (25405-1-AP, Proteintech), anti-BRCA1 (22362-1-AP, Proteintech), anti-SUZ12 (20366-1-AP, Proteintech), anti-FANCM (12954-1-AP, Proteintech), anti-ZO-1 (21773-1-AP, Proteintech), anti-Claudin 1 (13050-1-AP, Proteintech), anti-Occludin (66378-1-Ig, Proteintech), anti-β-Actin (4967S, Cell Signaling Technology), and anti-GAPDH (2118S, Cell Signaling Technology). Secondary antibodies used included anti-mouse conjugated to horseradish peroxidase (A2304, Sigma-Aldrich) and anti-rabbit conjugated to horseradish peroxidase (NA9340, GE Healthcare) for western blotting; anti-rabbit conjugated to Alexa Fluor 568 (A11011) and anti-mouse conjugated to Alexa Fluor 488 (A21202) were from Life Technology for confocal microscopy.

The Catalase Activity Assay Kit was purchased from Abcam (ab83464). DSS (160110) was from MP Biomedicals (Shanghai) Co. Ltd. MitoSox Red mitochondrial superoxide indicator was from Thermo Fisher Scientific (M3608). The 8-OHdG ELISA Kit was from Abcam (ab201734).

### Statistical analysis

All data were expressed as the mean ± standard derivation. Differences between two groups were compared by the Mann-Whitney U test or Student’s *t*-test. Multiple group comparisons were made by the Kruskal-Wallis test or one-way analysis of variance followed by Tukey’s *t*-test. *P* values less than 0.05 were considered statistically significant. See other details in the supplementary experimental procedures.

## Data Availability

The authors confirm that the data supporting the findings of this study are available within the article and the raw sequencing results can be accessed with the accession number GSE243405.

## SUPPLEMENTAL MATERIAL

Figure S1 msystems.00206-24-s0001.tif Identification of host-induced *H. pylori*-specific stress responses using Dual RNA-Seq, related to Fig. 1.

Supplemental material msystems.00206-24-s0002.docx Supplemental materials and methods and legend to Fig. S1.

Table S1 msystems.00206-24-s0003.xlsx Primers.

ASM does not own the copyrights to Supplemental Material that may be linked to, or accessed through, an article. The authors have granted ASM a non-exclusive, world-wide license to publish the Supplemental Material files. Please contact the corresponding author directly for reuse.
