# Supplementary figures and images for: Dual RNA sequencing of Helicobacter pylori and host cell transcriptomes reveals ontologically distinct host-pathogen interaction
Source: mSystems. 2024 Mar 22;9(4):e00206-24. doi: 10.1128/msystems.00206-24 (PMC11019886; doi:10.1128/msystems.00206-24)

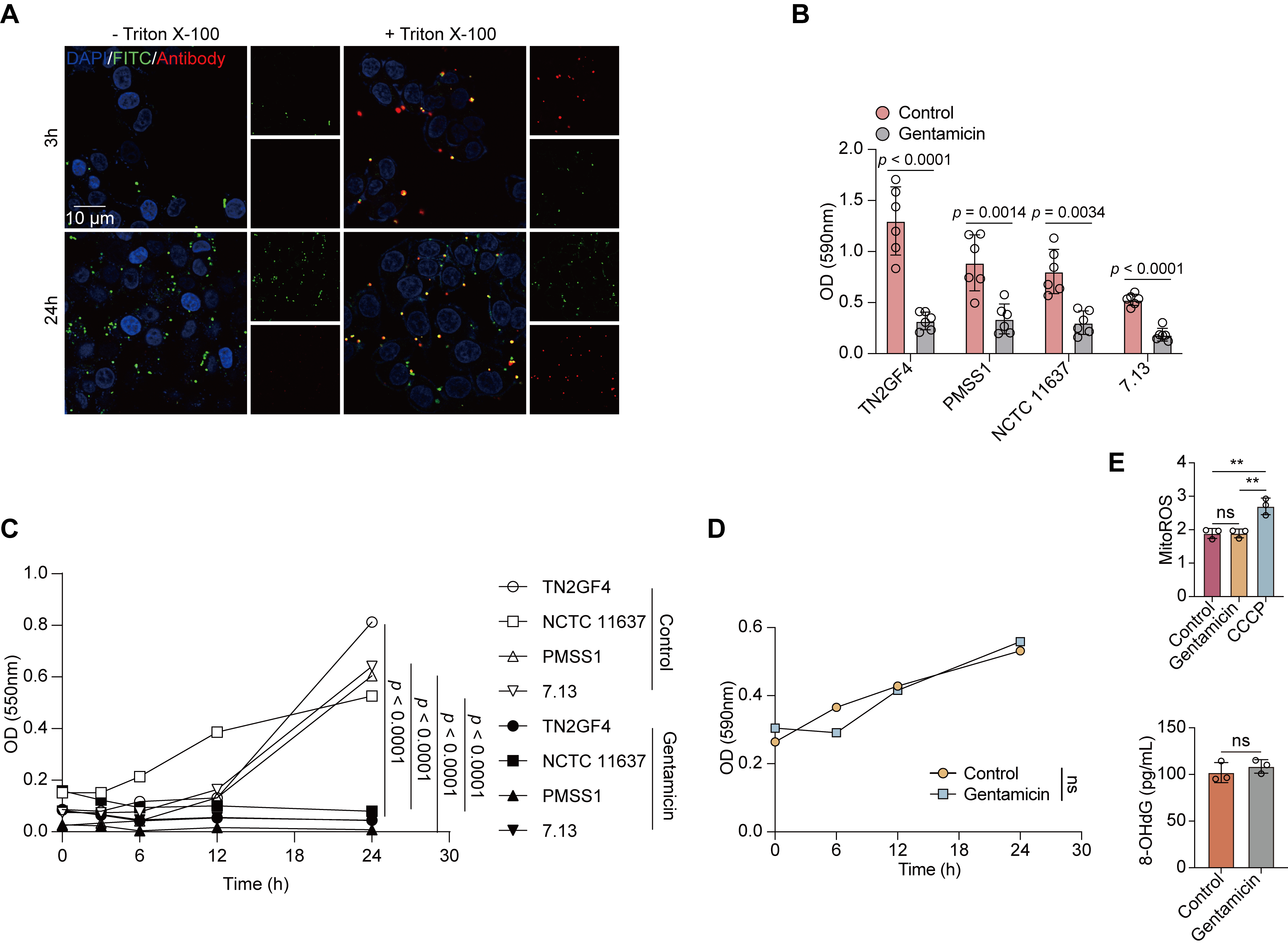

Supplement: Figure S1 — Identification of host-induced H. pylori-specific stress responses using Dual RNA-Seq, related to Fig. 1. [file msystems.00206-24-s0001.tif]
