# Supplementary material for: Dual RNA sequencing of Helicobacter pylori and host cell transcriptomes reveals ontologically distinct host-pathogen interaction
Source: mSystems. 2024 Mar 22;9(4):e00206-24. doi: 10.1128/msystems.00206-24 (PMC11019886; doi:10.1128/msystems.00206-24)
Supplement: Supplemental material — s and methods and legend to Fig. S1. [file msystems.00206-24-s0002.docx]

**Supplemental materials and methods**

*RNA Extraction, strand-specific library construction and sequencing*

GES-1 cells were infected with WT or *cagA*-mutated *H. pylori* TN2GF4 strain (MOI 100) for 3 h, then exposed to gentamicin (100 μg/ml) for 12 or 24 h to eliminate the extracellular bacteria. Total RNA was extracted using Trizol reagent kit (Invitrogen, Carlsbad, CA,USA) according to the manufacturer’s protocol. RNA quality was assessed on an Agilent 2100 Bioanalyzer (Agilent Technologies, Palo Alto, CA, USA) and checked using RNase free agarose gel electrophoresis. After total RNA was extracted, eukaryotic mRNA was enriched by Oligo(dT) beads, while prokaryotic mRNA was enriched by removing rRNA Ribo-Zero^TM^ Magnetic Kit (Epicentre, Madison, WI, USA). The enriched mRNA was fragmented into short fragments by using fragmentation buffer and reverse transcribed into cDNA with random primers. Second-strand cDNA were synthesized by DNA polymerase I, RNase H, dNTP (dUTP instead of dTTP) and buffer. Next, the cDNA fragments were purified with QiaQuick PCR extraction kit(Qiagen, Venlo, The Netherlands), end repaired, A base added, and ligated to Illumina sequencing adapters. Then UNG (Uracil-N-Glycosylase) was used to digest the second-strand cDNA. The digested products were size selected by agarose gel electrophoresis, PCR amplified, and sequenced using Illumina Novaseq6000 (or other platforms) by Gene Denovo Biotechnology Co. (Guangzhou, China).

*Dual RNA-seqBioinformatics analysis*

1. *Filtering of Clean Reads*

Reads obtained from the sequencing machines included raw reads containing adapters or low quality bases which would affect the following assembly and analysis. Thus, to get high quality clean reads, reads were further filtered by fastp (version 0.18.0) (1). The parameters were as follows:

1. removing reads containing adapters;
2. removing reads containing more than 10% of unknown nucleotides (N);
3. removing low quality reads containing more than 50% of low quality (Q-value ≤ 20) bases.
4. *Alignment with Ribosome RNA (rRNA)*

Short reads alignment tool Bowtie2 (version 2.2.8) was used for mapping reads to ribosome RNA (rRNA) database (2). The rRNA mapped reads were then removed. The remaining reads were further used in assembly and analysis of transcriptome.

1. *Alignment with reference genome*

An index of the reference genome was built, and paired-end clean reads were mapped to the reference genome using HISAT2 (version 2.1.0) with “-rna-strandness RF” and other parameters set as a default (3).

1. *Transcripts Reconstruction*

The reconstruction of transcripts was carried out with software Stringtie (version 1.3.4) (4, 5), which together with HISAT2, allow biologists to identify new genes and new splice variants of known ones.

1. *Quantification of Transcripts Abundance*

Transcripts abundances were quantified by software StringTie in a reference-based approach. For each transcription region, a FPKM (fragment per kilobase of transcript per millio n mapped reads) value was calculated to quantify its expression abundance and variations, using RSEM software (6).

1. *Principal Component Analysis*

Principal component analysis (PCA) was performed with R package gmodels (http://www.r-project.org/) in this experience. PCA is a statistical procedure that converts hundreds of thousands of correlated variables (transcripts expression) into a set of values of linearly uncorrelated variables called principal components. PCA is largely used to reveal the structure/relationship of the samples/datas.

1. *Differentially expressed transcripts (DEGs) Analysis*

RNAs differential expression analysis was performed by DESeq2 (7) software between two different groups (and by edgeR (8) between two samples). The genes/transcripts with the parameter of false discovery rate (FDR) below 0.05 and absolute fold change ≥2 was considered differentially expressed genes/transcripts. Differentially expressed coding RNAs were then subjected to enrichment analysis of GO functions and KEGG pathways.

1. *Alternative Splicing Analysis*

rMATS (version 4.0.1) (9) (http://rnaseq-mats.sourceforge.net/index.html) was used to identify alternative splicing events and analyze differential alternative splicing events between samples. We identified AS events with a false discovery rate (FDR) <0.05 in a comparison as significant AS events.

The classification of alternative splicing is as follows:

• SE: skipped exon

• MXE: mutually exclusive exon

• A5SS: alternative 5’ splicesite

• A3SS: alternative 3’ splicesite

• RI: retained intron

*Quantitative PCR analysis RNA experiment*

Total RNA was extracted from sub-confluent (70 - 80%) cultured cells using Trizol reagent kit (Inviitrogen, Carlsbad, CA, USA) according to the manufacture’s protocol. Reverse transcription reaction was performed with 500 ng RNA sample using PrimeScript^TM^ RT Master Mix (perfect real time) (Takara Bio Inc.) according to the instructions suggested by manufacture. The mixture was incubated at 37 ℃ for 15 minutes for reverse transcription, and 85 ℃ for 5 seconds for heat inactivation of reverse transcriptase. The cDNA products were kept at -20 ℃ until being used for PCR amplification. Quantitative real-time PCR reaction was performed using SYBR premix (Takara Bio Inc.) according to the standard instructions. The specificity of primers was examined by the melting-curve detection of PCR products. Triplicates were set for one target gene in every one experiment. β-Actin was served as the internal control for total cDNA amount and quality. The relative expression levels of target genes were validated by the △△CT calculation method. All primers used in this study were provided in **Table S1**.

H. pylori *Colonization and Colitis Induction*

This study was approved by the Animal Studies Committee of Southern Medical University. Male C57BL/6 mice (4 weeks old) were purchased from Shanghai Model Organisms (Shanghai, China). The mice were allowed to adapt to the environment for one weeks without any treatment. Mice were randomly divided into three groups, including Control, DSS-treated alone (DSS), and *H. pylori*-infected & DSS-treated (*H. pylori* & DSS). In the *H. pylori* & DSS groups, five weeks old mice were orally infected with doses of 10^8^ colony-forming units *H. pylori* strain PMSS1 5 times within 2 weeks. At 4 weeks post-infection, to induce chronic colitis, the DSS and *H. pylori* & DSS groups were subjected to 3 cycles of 3% DSS (7 days/cycle), each separated by 1 week of regular water. The schematic diagram showing the treatment algorithm is presented in **Figure 5C**. To evaluate the severity of colitis, body weight, rectal bleeding, and stool consistency were observed to generate a disease activity index (DAI) score, as described previously (10). All mice were killed at 42 days after DSS administration. Mice stool samples were collected for subsequent shotgun metagenomic sequencing and absolute quantitative metabolomics. The colon was removed, and the length was measured. A fragment of the distal colon was fixed in 10% phosphate-buffered formalin for subsequent histologic and immunofluorescent analysis. The rest of the colon tissue was collected for further protein or mRNA extraction.

*Mice stool sample DNA extraction*

Mice stool sample DNA was extracted at Novogene Bioinformatics Technology (Beijing, China) using the SDS method. DNA was subsequently diluted to 1 ng/μl using sterile ddH_2_O, and its degradation degree and contamination were assessed on 1% agarose gels. DNA purity (OD260/OD280) was determined using the NanoDrop Microvolume Spectrophotometer (Thermo Fisher Scientific, USA). DNA concentration was measured using the Qubit^®^ dsDNA Assay Kit in Qubit^®^ 2.0 Fluorometer (Life Technologies, Carlsbad, CA, USA).

*Metagenomic shotgun sequencing and bioinformatic analysis*

The raw data was in FASTQ format. Reads were trimmed and filtered using Trimmomatic (v0.36) (11). Host pollution control was needed if the DNA was extracted from host-related environment. The post-filtered pair-end reads were aligned against the host genome using bowtie2 (v2.2.9) (2) and the aligned reads were discarded. Metagenome assembly was performed using MEGAHIT (v1.1.2) (12) after getting valid reads. Use gaps inside scaffold as breakpoint to interrupt the scaffold into new contigs (Scaftig), and these new Scaftig with length ≥ 200 bp (or 500 bp) of were retained. ORF prediction of assembled scaffolds using prodigal (v2.6.3) (13) was performed and translated into amino acid sequences. The non-redundant gene sets were built for all predicted genes using CDHIT (v4.6.7) (14). The clustering parameters were 95% identity and 90% coverage. The longest gene was selected as representative sequence of each gene set. Clean reads of each sample were aligned against the non-redundant gene set (95% identity) use bowtie2 (v2.2.9), and the abundant information of the gene in the corresponding sample was counted. The gene set representative sequence (amino acid sequence) was annotated with NR, KEGG, COG, SWISSPROT, GO database with an e-value of 1e^-5^. The taxonomy of the species was obtained as a result of the corresponding taxonomy database of the NR Library, and the abundance of the species was calculated using the corresponding abundance of the genes. In order to construct the abundance profile on the corresponding taxonomy level, abundance statistics were performed at each level of Domain, Kingdom, Phylum, Class, Order, Family, Genus, Species. The PCA analysis and plotting of the abundance spectrum of the species or functional abundance spectrum were carried out using R software (v3.2.0).

*Immunofluorescence*

For immunofluorescence staining of mouse colon sections, slides were incubated with primary antibody followed by a secondary fluorescent antibody and then 4', 6-diamidino-2-phenylindole (DAPI) to stain cell nuclei. Sections were evaluated using laser scanning confocal microscopy (Olympus FV1000). The immunofluorescence intensity was quantitatively measured using the ImageJ program.

*Histopathological assessment*

For pathological assessment, the H&E stained sections were evaluated by a blinded pathologist following the criteria of histological score previously reported(15). The colitis score (maximum = 8) was the sum of the following two features: Epithelium (0: Normal morphology; 1: Loss of goblet cells, 2: Loss of goblet cells in large areas; 3: Loss of crypts; 4: Loss of crypts in large areas), and infiltration (0: No infiltrate; 1: Infiltrate around crypt basis; 2: Infiltrate reaching to lamina muscularis mucosae; 3: Extensive infiltration reaching the lamina muscularis mucosae and thickening of the mucosa with abundant edema; 4: Infiltration of the lamina submucosa).

Mitochondrial superoxide detection

Mitochondrial superoxide is detected by MitoSox Red mitochondrial superoxide indicator (Invitrogen, Carlsbad, CA). Briefly, GES-1 cells were cultured in 35mm 1.5 glass bottom dishes containing gentamicin free media overnight. The media were aspirated at second day. The dishes were filled with media containing 100 μg/ml gentamicin. The control cells were cultured in media without gentamicin addition. All cells were cultured for 24 hours and stained with 5 μM MitoSox Red reagent working solution for 10 minutes at 37℃. The cells were washed 3 times with PBS solution and obtained fluorescence by Olympus FV1000 fluorescent microscope. More than 20 pictures were taken in different areas each dish. The immunofluorescence intensity was quantitatively measured from three independent experiments using the ImageJ program.

*DNA oxidative damage examination*

GES-1 cells were sub-cultured in triple on 96-well cell culture plate and cultured in media containing 100 μg/ml gentamicin for 24 hours. Cells cultured in gentamicin free media were used as the controls. DNA oxidative damage is examined by DNA Damage Competitive ELISA Kit to determine the formation of 8-OHdG according to the manufacturer’s protocol.

*Western blots*

Tissues or cells were lysed in radioimmunoprecipitation assay buffer containing 0.5% cholic acid, 0.1% sodium dodecyl sulfate, 2 mmol/L ethylenediaminetetraacetic acid, 1% Triton X-100, 10% glycerol, 1.0 mM phenylmethylsulfonyl fluoride and 1 µg/mL aprotinin. After sonication for 30 sec on ice and centrifuging for 20 min at 12,000 rpm at 4°C, the supernatant was collected, and protein concentration was determined by protein assay kit (Bio-Rad), using bovine serum albumin (BSA) as the standard. 20 μg of protein samples were resolved on SDS polyacrylamide gel electrophoresis (SDS-PAGE). Proteins were blotted onto nitrocellulose membranes (Bio-Rad). Non-specific binding was blocked in 5% blocking buffer (5% non-fat dry milk, 150 mmol/L Tris-HCl, pH 7.4, 50 mmol/L NaCl, 0.05% Tween 20) for 1 hr, and membranes were incubated overnight at 4°C with indicated with primary antibody at a 1:1000 dilution. The membranes were then incubated for 1hr with horseradish peroxidase-coupled secondary antibody at room temperature. Chemiluminescent signals were then developed with LumiGLO reagent (Cell Signaling Technology) and exposed on X-ray film (Fuji Photo Film Co., Ltd.).

**Reference**

1. Chen S, Zhou Y, Chen Y, Gu J. 2018. fastp: an ultra-fast all-in-one FASTQ preprocessor. Bioinformatics 34:i884-i890.

2. Langmead B, Salzberg SL. 2012. Fast gapped-read alignment with Bowtie 2. Nat Methods 9:357-9.

3. Kim D, Langmead B, Salzberg SL. 2015. HISAT: a fast spliced aligner with low memory requirements. Nat Methods 12:357-60.

4. Trapnell C, Williams BA, Pertea G, Mortazavi A, Kwan G, van Baren MJ, Salzberg SL, Wold BJ, Pachter L. 2010. Transcript assembly and quantification by RNA-Seq reveals unannotated transcripts and isoform switching during cell differentiation. Nat Biotechnol 28:511-5.

5. Pertea M, Pertea GM, Antonescu CM, Chang TC, Mendell JT, Salzberg SL. 2015. StringTie enables improved reconstruction of a transcriptome from RNA-seq reads. Nat Biotechnol 33:290-5.

6. Li B, Dewey CN. 2011. RSEM: accurate transcript quantification from RNA-Seq data with or without a reference genome. BMC Bioinformatics 12:323.

7. Love MI, Huber W, Anders S. 2014. Moderated estimation of fold change and dispersion for RNA-seq data with DESeq2. Genome Biol 15:550.

8. Robinson MD, McCarthy DJ, Smyth GK. 2010. edgeR: a Bioconductor package for differential expression analysis of digital gene expression data. Bioinformatics 26:139-40.

9. Shen S, Park JW, Lu ZX, Lin L, Henry MD, Wu YN, Zhou Q, Xing Y. 2014. rMATS: robust and flexible detection of differential alternative splicing from replicate RNA-Seq data. Proc Natl Acad Sci U S A 111:E5593-601.

10. Xu X, Wang Y, Wei Z, Wei W, Zhao P, Tong B, Xia Y, Dai Y. 2017. Madecassic acid, the contributor to the anti-colitis effect of madecassoside, enhances the shift of Th17 toward Treg cells via the PPARgamma/AMPK/ACC1 pathway. Cell Death Dis 8:e2723.

11. Bolger AM, Lohse M, Usadel B. 2014. Trimmomatic: a flexible trimmer for Illumina sequence data. Bioinformatics 30:2114-20.

12. Li D, Liu CM, Luo R, Sadakane K, Lam TW. 2015. MEGAHIT: an ultra-fast single-node solution for large and complex metagenomics assembly via succinct de Bruijn graph. Bioinformatics 31:1674-6.

13. Hyatt D, Chen GL, Locascio PF, Land ML, Larimer FW, Hauser LJ. 2010. Prodigal: prokaryotic gene recognition and translation initiation site identification. BMC Bioinformatics 11:119.

14. Li W, Jaroszewski L, Godzik A. 2001. Clustering of highly homologous sequences to reduce the size of large protein databases. Bioinformatics 17:282-3.

15. Erben U, Loddenkemper C, Doerfel K, Spieckermann S, Haller D, Heimesaat MM, Zeitz M, Siegmund B, Kuhl AA. 2014. A guide to histomorphological evaluation of intestinal inflammation in mouse models. Int J Clin Exp Pathol 7:4557-76.

**Supplemental figure legends**

**Figure S1. Identification of host-induced *H. pylori*-specific stress responses using Dual RNA-Seq, related to Figure 1. (A)** FITC-labeled *H. pylori* TN2GF4 strain was co-cultured with GES-1 cells (MOI 100) for 3 or 24 h, followed by 0.1% Triton X-100 or PBS treatment. Cells were then stained to visualize *H. pylori* using antibody (Red). Representative images were showed. Scale bar = 10 μm. **(B)** *H. pylori* TN2GF4, PMSS1, NCTC 11637 or 7.13 strains were exposed to gentamicin (100 μg/ml) for 24 h. Biofilm formation was determined by 1% crystal violet staining at OD_590nm_. **(C)** *H. pylori* TN2GF4, PMSS1, NCTC 11637 or 7.13 strains were exposed to gentamicin (100 μg/ml) or PBS for indicated timepoints. Bacterial growth was measured at OD_550nm_. **(D)** GES-1 cells were treated with gentamicin (100 μg/ml) or PBS for indicated timepoints. Cell growth was determined by MTT assay at OD_590nm_. **(E)** GES-1 cells were treated with gentamicin (100 μg/ml) or CCCP for 24 h. (Upper) The production of mitochondrial superoxide was measured by the MitoSox Red reagent. CCCP was used as the positive control. (Down) 8-OHdG concentration (pg/mL) in the media was measured by DNA Damage Competitive assay kit. All the quantitative data were presented as means ± S.D. from three independent experiments. **P* < 0.05; ***P* < 0.01; ****P* < 0.001.
